# Basal Immunoglobulin Signaling Actively Maintains Developmental Stage in Immature B Cells

**DOI:** 10.1371/journal.pbio.0030082

**Published:** 2005-03-08

**Authors:** Lina E Tze, Brian R Schram, Kong-Peng Lam, Kristin A Hogquist, Keli L Hippen, Jiabin Liu, Susan A Shinton, Kevin L Otipoby, Peter R Rodine, Amanda L Vegoe, Manfred Kraus, Richard R Hardy, Mark S Schlissel, Klaus Rajewsky, Timothy W Behrens

**Affiliations:** **1**Center for Immunology, University of Minnesota Medical SchoolMinneapolis, MinnesotaUnited States of America; **2**Institute of Molecular and Cell BiologySingapore; **3**Fox Chase Cancer Center, PhiladelphiaPennsylvaniaUnited States of America; **4**Center for Blood Research, Harvard Medical SchoolBoston, MassachusettsUnited States of America; **5**Department of Molecular and Cell Biology, University of CaliforniaBerkeley, CaliforniaUnited States of America; Scripps Research InstituteUnited States of America

## Abstract

In developing B lymphocytes, a successful V(D)J heavy chain (HC) immunoglobulin (Ig) rearrangement establishes HC allelic exclusion and signals pro-B cells to advance in development to the pre-B stage. A subsequent functional light chain (LC) rearrangement then results in the surface expression of IgM at the immature B cell stage. Here we show that interruption of basal IgM signaling in immature B cells, either by the inducible deletion of surface Ig via Cre-mediated excision or by incubating cells with the tyrosine kinase inhibitor herbimycin A or the phosphatidylinositol 3-kinase inhibitor wortmannin, led to a striking “back-differentiation” of cells to an earlier stage in B cell development, characterized by the expression of pro-B cell genes. Cells undergoing this reversal in development also showed evidence of new LC gene rearrangements, suggesting an important role for basal Ig signaling in the maintenance of LC allelic exclusion. These studies identify a previously unappreciated level of plasticity in the B cell developmental program, and have important implications for our understanding of central tolerance mechanisms.

## Abstract 2

Gene rearrangement is a hallmark of B cell maturation. By interrupting basal cell signaling through the rearranged IgM receptor, immature B cells "back-differentiate" to an earlier stage in their development

## Introduction

B lymphocytes follow a highly ordered program of development in the bone marrow (BM), beginning with the commitment of lymphoid progenitors to the B lineage and the somatic recombination of heavy chain (HC) immunoglobulin (Ig) alleles [1]. Following an initial diversity (D_H_) to joining (J_H_) gene segment rearrangement, usually on both alleles, pro-B cells then rearrange one of many upstream variable (V_H_) region segments to the D-J_H_ segment, creating the V(D)J joint. These rearrangements require the action of the lymphoid-specific recombination activating genes Rag1 and Rag2, together with a number of ubiquitously expressed DNA repair proteins [2]. Cells with a productive protein-encoding HC rearrangement express HC together with invariant surrogate Ig light chains VpreB and lambda 5 (λ5), and then undergo clonal expansion before efficient initiation of rearrangements at light chain (LC) loci (kappa, κ, or lambda, λ) [3]. A productive LC rearrangement results in the cell surface expression of IgM, which defines the immature B cell stage (IgM^+^IgD^−^).

Due to the stochastic nature of V(D)J recombination, B cells express an extremely diverse Ig receptor repertoire (more than 10^9^ specificities). To reduce the potential for autoimmune antibody responses, cells bearing strongly self-reactive Ig receptors are tolerized, either by clonal deletion, functional inactivation through the induction of anergy, or by receptor editing where new LC rearrangements revise the antigen (Ag) specificity of the receptor [4,5]. The maintenance of tolerance also requires that individual B cells express a single Ig HC and LC, since cells bearing multiple receptors could have significant autoimmune potential. In addition, cells bearing receptors in which the two antibody binding sites are not identical would have a reduced ability to bind certain antigens, which could, in turn, compromise downstream antibody effector functions such as complement activation [6].

The process by which cells express a single receptor is called allelic exclusion [3], with a functional Ig rearrangement likely providing a “stop” signal that blocks further rearrangements. In general, the mechanisms that initiate and maintain allelic exclusion are not well understood. HC allelic exclusion requires the expression of a functional membrane-bound HC protein, since mice lacking the Cμ transmembrane domain show a complete block in B cell development at the pro-B stage, and B cells fail to establish HC allelic exclusion [7]. HC allelic exclusion also requires the Ig receptor-associated signaling proteins Igα and Igβ [8,9,10,11]. Less is known about the signaling requirements for LC allelic exclusion, where the situation is complex due to the presence of two κ and two λ alleles and the potential for multiple rearrangements at each locus. LC receptor editing occurs in immature B cells with self-reactive Ig receptors, and continues until a suitable receptor is formed, whereupon further rearrangements are suppressed. Recent studies indicate that receptor editing at LC loci is a common theme in normal B cell development, occurring in approximately 20% or more of B cells during their maturation [12].

Despite the importance of receptor editing in shaping the B cell immune repertoire, our understanding of the mechanisms that drive editing are rudimentary. It is clear that Rag proteins can be re-induced in immature B cells following B cell receptor (BCR) crosslinking by self-antigen, and that this can lead to new rearrangements at LC loci [13,14]. The prevailing view is that positive signaling through crosslinked BCRs drives the editing response. However, in experiments investigating LC receptor editing responses to soluble self-antigen, we found an inverse relationship between levels of surface IgM and LC editing (i.e., low levels of IgM associated with high levels of editing) [15]. These data were consistent with the hypothesis that tonic signals provided by surface BCRs are important for suppressing editing responses in immature B cells. The experiments here were designed to examine this possibility, and the results suggest that basal signaling from the BCR is critical for immature B cells to suppress Rag expression and, surprisingly, to maintain developmental stage.

## Results

### Model for Inducible Deletion of Surface IgM in Immature B Cells

For the initial series of experiments, we used transgenic mice specifically generated to examine the consequences of inducible deletion of the BCR on B cell function [16]. These animals carry a “floxed” B1-8 HC knock-in allele (B1-8f, flanked by LoxP recombination sites), a 3-83κ LC knock-in allele (3-83κ) [17], and an interferon (IFN)-inducible Cre recombinase transgene (Mx-Cre) [18]. In previous experiments, in vivo treatment of mice carrying these transgenes with type I IFN led to the induction of Cre, followed by the efficient deletion of the B1-8f HC and loss of BCR surface expression. These studies demonstrated that the survival of mature B cells was critically dependent on basal signaling by the BCR [16].

**Figure 1:**
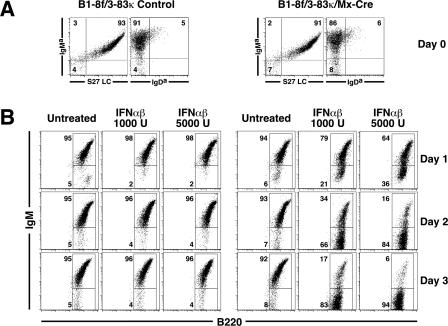
Inducible Cre-Mediated Deletion of the B1-8f HC Allele Leads to Loss of Surface Ig from Immature B Cells (A) Flow cytometry showing surface phenotype of B1-8f/3-83κ and B1-8f/3-83κ/Mx-Cre BM after 5-d IL-7 culture. (B) Flow cytometric analysis of B1-8f/3-83κ and B1-8f/3-83κ/Mx-Cre BM culture cells incubated with IFNαβ (1,000 or 5,000 units/ml), in the absence of IL-7, for 1, 2, or 3 d. The cell populations shown are gated on lymphocytes by forward and side scatter, and then for B220. At the end of the 5-d IL-7 culture, more than 90% of the cells were viable and B220^+^. The numbers shown indicate the percentage of gated cells.

Bone marrow from B1-8f/3-83κ/Mx-Cre and control B1-8f/3-83κ mice was incubated in vitro with interleukin-7 (IL-7), a potent growth factor for early B cell progenitors, for 5 d. At the end of the culture period, flow cytometry revealed an expanded population of IgM^+^IgD^−^ immature B cells that expressed the B1-8 IgM^a^ allotype and the 3-83κ LC [recognized by the S27 antibody (Figure 1A)]. All of the IgM^a^ allotype-positive cells also stained with an anti-B1-8 idiotype antibody (data not shown). Additional cell surface profiling of these cells is presented in box 4, A small population of B220^+^ cells was negative for the BCR in both cultures.

Cells were then washed and incubated with either 1,000 or 5,000 units/ml of IFN αβ for 1, 2, and 3 d, in the absence of IL-7. There was efficient IFN-dependent deletion of surface IgM in B1-8f/3-83κ/Mx-Cre B cells that was apparent by day 1 and was approximately 90% complete at day 3 (Figure 1B). Deletion was more efficient with 5,000 units/ml of IFNαβ than with 1,000 units/ml. Control experiments demonstrated that the cells lacking IgM receptor derived from the IgM^hi^ population following Cre-mediated deletion of HC (described in detail below).

### Microarrays to Evaluate Gene Expression in Cells Undergoing Inducible Deletion of IgM

**Figure 2:**
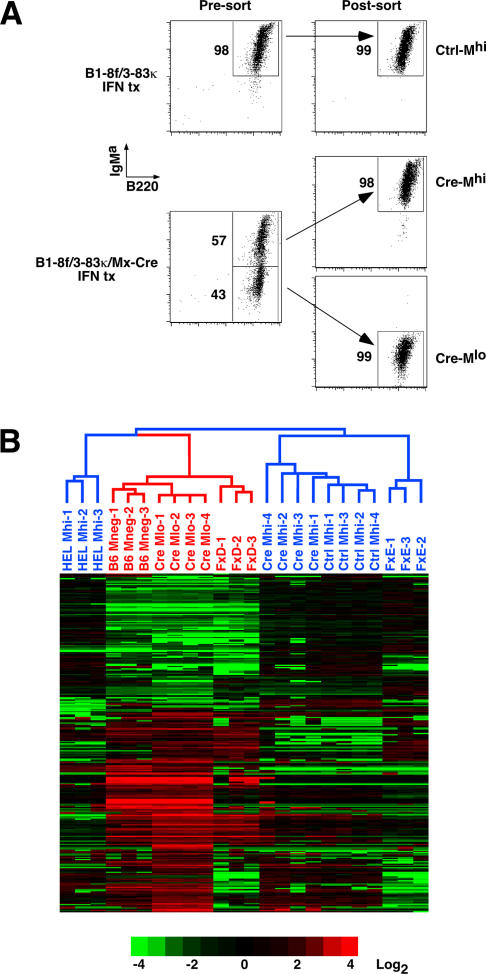
Microarray Gene Expression Analysis Demonstrates Co-Clustering of Cre-Deleted IgM^lo^ Cells with IgM^−^ Cell Populations (A) FACS sorting strategy for Ctrl-M^hi^, Cre-M^hi^, and Cre-M^lo^ immature B cells incubated with IFNαβ (1,000 units/ml) for 2 d. The numbers shown indicate the percentage of gated cells. (B) Affymetrix microarrays were used to identify genes differentially expressed between IFN-treated Ctrl-M^hi^ and Cre-M^lo^ cells. Analysis identified 327 transcripts that met the following criteria: 2-fold or greater change in mean expression level, a more than 200-unit difference in mean expression values, and Student's t-test *p* < 0.01. Individual expression values for each gene were divided by the mean of expression levels for three control IgM^+^ cell populations: IFN-treated Ctrl-M^hi^; IgM^hi^ cells sorted from 5-d IL-7 HEL-lg BM cultures (HEL-M^hi^); and sorted from normal Balb/c BM (FxE). Other populations included Cre-M^hi^, Hardy Fraction D pre-B cells (FxD) sorted from normal Balb/c BM, and lgM^−^ cells sorted from 5-d IL-7 cultures of control B6 BM (B6-M^−^). Data were transformed into log_2_ space, and represent fold-differences relative to the IgM^+^ cell populations (see scale bar). Data from 293 transcripts (duplicates and all but four representative Ig HC and LC transcripts removed) were clustered and visualized using CLUSTER and TREEVIEW [51]. Red represents genes expressed at higher levels, while green represents genes expressed at lower levels, than the mean of IgM^+^ cells. Each column represents an individual sorted cell population.

IFN-treated B1-8f/3-83κ IgM^hi^ (Ctrl-M^hi^), B1-8f/3-83κ/Mx-Cre IgM^hi^ (Cre-M^hi^), and B1-8f/3-83κ/Mx-Cre IgM-deleted (Cre-M^lo^) cell populations were sorted from 2-d IFN cultures (Figure 2A), total RNA was isolated, and biotinylated cRNA probes were generated (see Materials and Methods). Probes were hybridized with Affymetrix U74A mouse GeneChip microarrays, which represent approximately 11,000 transcripts, and expression levels were quantified using Affymetrix MicroArray Suite 5.0 software. The data from each array were scaled to correct for minor differences in overall array hybridization intensity, and analyzed to identify genes differentially expressed between Ctrl-M^hi^ and Cre-M^lo^ cells.

Analysis of the array data confirmed that Ig HC gene expression dropped dramatically in the Cre-M^lo^ cell population (Table 1; two representative probesets are shown), while κ LC gene expression showed a significant approximately 2-fold up-regulation in Cre-M^lo^ cells. Strikingly, Cre-M^lo^ cells had elevated levels of mRNA for Rag1 and Rag2, the DNA repair enzyme Ku70, the surrogate light chains VpreB and λ5, as well as the terminal deoxynucleotidyl transferase (TdT), an enzyme thought to play a particularly important role in the generation of junctional diversity of B cell HC alleles.

**Table 1:**
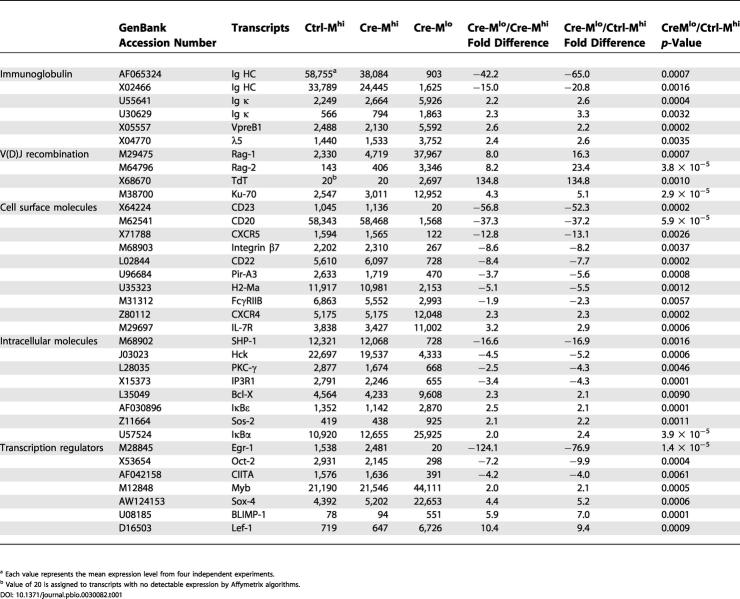
Representative Gene Expression in Cells Undergoing Cre-Mediated Deletion of IgM

^a^ Each value represents the mean expression level from four independent experiments

^b^ Value of 20 is assigned to transcripts with no detectable expression by Affymetrix algorithms

### Surface BCR Expression Is Required to Maintain B Cell Developmental Stage

Further examination of the microarray data revealed a large number of genes that were differentially expressed between Ctrl-M^hi^ and Cre-M^lo^ cells. A total of 327 transcripts met the following criteria: a 2-fold or greater difference in expression levels, expression value difference more than 200 units, and T-test *p* < 0.01. In order to better understand the significance of the gene expression changes observed between Ctrl-M^hi^ and Cre-M^lo^ cells, we prepared cRNA probes and performed microarray analyses on several additional sorted, control cell populations: (a) IgM^−^ pro-B cells from 5-d IL-7 cultures of BM from nontransgenic C57Bl/6J mice (B6-M^−^); (b) IgM^hi^ cells from 5-d IL-7 BM cultures from hen egg lysozyme (HEL) Ig transgenic mice [19] (HEL-M^hi^); (c) Hardy fraction (Fx) [20] “D” pre-B cells (B220^+^CD43^−^IgM^−^) from normal Balb/c BM (FxD); and (d) Fx “E” immature B cells (B220^+^IgM^+^IgD^−^), also from normal Balb/c BM (FxE).

To visualize the microarray data, expression values for each transcript were first converted to fold-difference ratios by dividing each value by the mean expression level for the IgM^hi^ cell populations (Ctrl-M^hi^, HEL-M^hi^, and FxE). These ratios were then converted into log_2_ space, and the data were analyzed by unsupervised hierarchical clustering.

Visualization of the data showed that approximately 60% of the genes were overexpressed (red in Figure 2B) in Cre-M^lo^ relative to Ctrl-M^hi^ or Cre-M^hi^ cells, while approximately 40% were down-regulated (green in Figure 2B). Strikingly, the Cre-M^lo^ cells clustered with normal pre-B cells (FxD) and with surface IgM-negative cells (B6-M^−^), rather than with the IgM^+^ cell populations. This suggested the possibility that Cre-M^lo^ cells had differentiated back to an earlier stage in B cell development as a consequence of losing IgM receptor expression.

**Figure 3:**
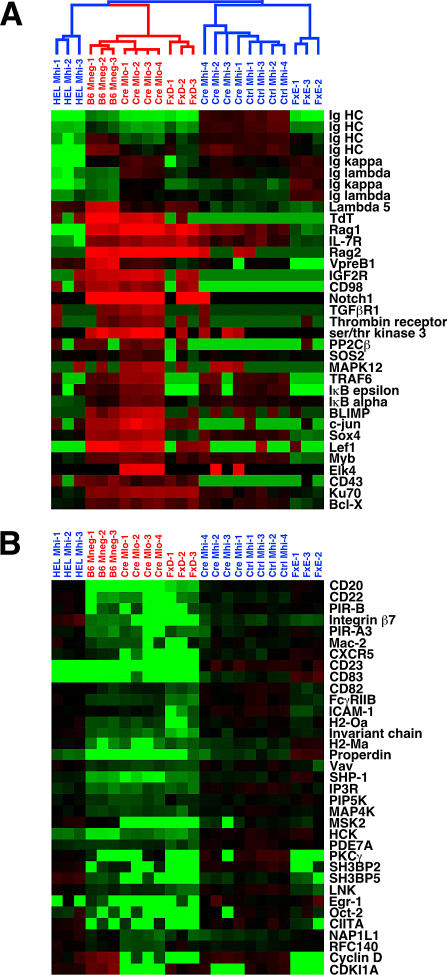
Genes Differentially Expressed between Ctrl-M^hi^ and Cre-M^lo^ Cell Populations Shown are representative genes that were generally either (A) up-regulated or (B) down-regulated in Cre-M^lo^ cells compared with Ctrl-M^hi^ cells. See Figure 2 legend for details.

Of the genes that were differentially expressed between Ctrl-M^hi^ and Cre-M^lo^, many are developmentally regulated during normal B cell differentiation (Figure 3 and Table 1). In addition to the gene expression changes noted above, Cre-M^lo^ cells showed up-regulated mRNA levels for CD43 and the IL-7 receptor (IL-7R), genes selectively expressed in pro-B cells. Cre-M^lo^ cells also showed elevated levels of mRNA for the cell surface proteins insulin-like growth factor II receptor, CD98, Notch1, transforming growth factor β receptor, and the thrombin receptor; the intracellular signaling molecules serine-threonine kinase 3, protein phosphatase 2Cβ, Son of Sevenless 2, and mitogen-activated protein kinase; and transcription factors such as B lymphocyte-induced maturation protein (BLIMP), c-jun, sex determining region Y-box 4, lymphoid enhancer factor 1, myb, and elk4. Many of the genes expressed at high levels in Cre-M^lo^ cells were also expressed at elevated levels in FxD and B6-M^neg^ cell populations, compared with the lower expression levels found in the various IgM^+^ populations (Figure 3).

A similar situation held for genes that were down-regulated in Cre-M^lo^ cells compared to Ctrl-M^hi^. Transcripts for a large number of surface markers that characterize mature B cells (CD20, CD22, CD23, CD82, CD83, integrin β7, paired-Ig-like receptor-A3 and -B, Mac-2, CXCR5, Fcγ receptor IIB, and ICAM-1/CD54) were expressed at significantly lower levels in Cre-M^lo^ than in Ctrl-M^hi^ or Cre-M^hi^ cells. Other genes down-regulated in Cre-M^lo^ cells included those encoding class II major histocompatibility complex (MHC) molecules, intracellular signaling molecules (e.g., Vav, Src homology 2 phosphatase-1 (SHP-1), the inositol 1,4,5-trisphosphate receptor, phosphatidylinositol phosphate 5-kinase, MAP4K, mitogen- and stress-activated protein kinase 2, hemopoietic cell kinase, phosphodiesterase 7A, and protein kinase Cγ), and adaptor proteins (e.g., SH3-domain binding protein-2 and -5, and LNK). Finally, the mRNAs for several interesting nuclear proteins were also down-regulated in Cre-M^lo^ cells, including the transcription factors Oct-2, class II transactivator (CIITA), and early growth response 1, as well as the cell cycle regulators cyclin D2 and cyclin-dependent kinase inhibitor 1a. Nearly all of the down-regulated genes in Cre-M^lo^ cells were also expressed at low levels in B6-M^−^ pro-B cells and FxD cells. From these data, we conclude that the B cell developmental program has shifted into reverse in immature B cells that have undergone inducible loss of surface IgM expression.

### Cre-M^lo^ Cells Derive from Cre-M^hi^ Cells

**Figure 4:**
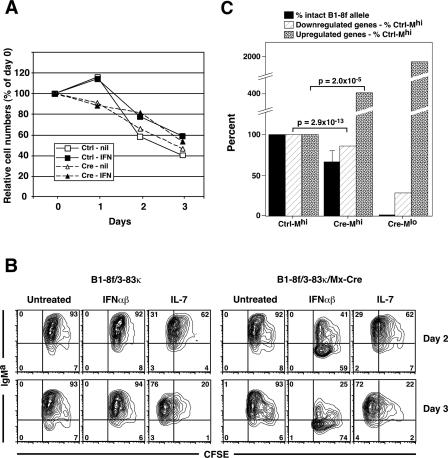
Gene Expression Phenotype of Cre-M^hi^ Cell Populations Is Intermediate between Ctrl-M^hi^ and Cre-M^lo^ Populations (A) Viable cell counts were performed in control B1-8f/3-83κ and B1-8f/3-83κ/Mx-Cre cell populations at the initiation of culture with medium alone (nil) or with IFNαβ (IFN) 1,000 units/ml, and daily for 3 d. Data are presented as percent of the day 0 cell numbers, and represent 4–7 experiments for each population. Standard errors were less than 10%, and are not shown. (B) BM cells were cultured for 5 d in IL-7, and were then labeled with CFSE and incubated with 1,000 units/ml IFNαβ or, as a proliferation control, 16 ng/ml IL-7 for an additional 3 d. Flow cytometric analysis of CFSE dye dilution of B220^+^ cells indicated no significant proliferation of IFN-treated cell populations. Numbers represent percent of gated cells. (C) A PCR-based assay was used to quantitate the extent of B1-8f deletion in Ctrl-M^hi^, Cre-M^hi^, and Cre-M^lo^ populations. Ctrl-M^hi^ cells contained 100% intact B1-8f alleles, Cre-M^lo^ cells were 100% deleted, and Cre-M^hi^ cells had 34 ± 16% average deletion of the B1-8f allele. Expression values for the Ctrl-M^hi^ transcripts (significantly different between Ctrl-M^hi^ and Cre-M^lo^) were normalized to 1, and relative expression levels of transcripts up- (*n* = 184) and down-regulated (*n* = 143) in the three populations were calculated. In both groups of genes, Cre-M^hi^ cells showed intermediate levels of gene expression between Ctrl-M^hi^ and Cre-M^lo^, indicating that Cre-M^lo^ cells originated from the Cre-M^hi^ population.

It was important to rule out the possibility that the back-differentiation observed in Cre-M^lo^ cells might be an artifact of selective expansion and/or survival of IgM^−^ cells that were present at the initiation of the IFN cultures. Cell counts throughout the culture period revealed that the overall number of viable cells was not significantly different between control and BCR-deleting populations (Figure 4A). This was confirmed by similar annexin V and 7-amino-actinomycin D (7-AAD) flow cytometric staining profiles (box 4 (nd data not shown). CFSE [5(6)-carboxyfluorescein diacetate succinimidyl ester] labeling indicated that cells cultured with IFNαβ were not proliferating at significant levels (Figure 4B). We also isolated highly purified populations of IgM^+^ immature B cells prior to incubation with IFN, and found that these cells similarly underwent the back-differentiation response (Figure S1. Thus, these experiments ruled out a selective expansion of pre-existing IgM^−^ cells in the IFN-treated B1-8f/3-83κ/Mx-Cre cultures.

Additional strong evidence that Cre-M^lo^ cells were derived from Cre-M^hi^ cells was the observation that Cre-M^hi^ cells showed an “intermediate” gene expression phenotype between Ctrl-M^hi^ and Cre-M^lo^ cell populations. As shown in Table 1, the level of transcript expression for most of the genes in Cre-M^hi^ cells was intermediate between Ctrl-M^hi^ and Cre-M^lo^ cell populations. Using a PCR-based assay (see Materials and Methods), we determined that about a third of the cells in the Cre-M^hi^ population (mean 34 ± 16%, range 14–48%) had undergone deletion of the floxed B1-8f allele (Figure 4C). When we compared the 327 transcripts that best discriminated Cre-M^lo^ from Ctrl-M^hi^ cells (see Figure 2B), the “intermediate” gene expression phenotype of Cre-M^hi^ cells was generally observed across the entire spectrum of genes (Figure 4C). The difference in gene expression between Cre-M^hi^ cells and the other populations was highly statistically significant (Figure 4C).

### Reversal in Differentiation Is Confirmed by Analysis of Cell Surface Proteins

**Figure 5:**
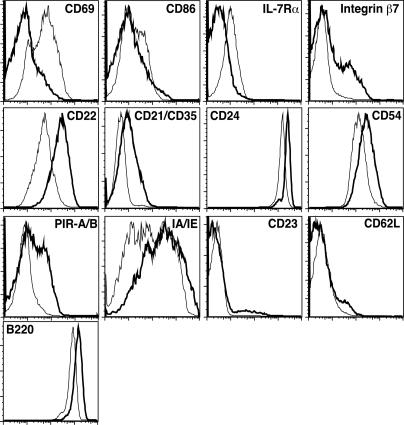
Protein Expression Confirms Reversal of Development in Immature B Cells Losing Surface IgM B1-8f/3-83κ control and B1-8f/3-83κ/Mx-Cre cell populations were harvested at the end of a 3- or 4-d culture with IFNαβ (3,000 units/ml), stained with mAbs for cell surface proteins, and analyzed by flow cytometry. Shown are the expression levels of B220-gated Ctrl-M^hi^ (thick line) and Cre-M^lo^ (thin line) cells at the end of the culture period. Essentially identical results were observed when Cre-M^hi^ and Cre-M^lo^ cells were compared (data not shown).

The changes in gene expression in Cre-M^lo^ cells were also reflected at the level of protein expression (Figure 5). After 3–4 d of culture, flow cytometry demonstrated that Cre-M^lo^ cells up-regulated surface expression of the activation antigens CD69 and CD86, as well as the IL-7R. Proteins down-regulated to varying degrees included B220, integrin β7, CD22, CD21/35, CD24, CD54, PirA/B, and Class II IA/IE. A few of the genes that showed differences at the mRNA level did not show measurable differences in protein expression (e.g., CD23). In nearly every case, the up- or down-regulation of surface protein expression mirrored the changes in mRNA expression. Importantly, the cells within each culture showed a similar cell surface phenotype, confirming that the back-differentiation was occurring across the entire population and not in just a minor subset. Additional control experiments supported the conclusion that back-differentiation of these cells was occurring in response to Cre-mediated loss of BCR expression, and not simply to elevated Cre levels (Figure S2 and S5).

### Herbimycin A and Wortmannin also Induce Back-Differentiation of Immature B Cells

To begin to explore the mechanisms by which basal IgM signaling maintains developmental stage, we first examined the influence of blocking tyrosine kinase signaling pathways downstream of the BCR with the inhibitor herbimycin A. For these experiments we used BM from mice carrying both a conventional Ig transgene (HEL-Ig) [19], and a bacterial artificial chromosome transgene containing green fluorescent protein (GFP) under the regulatory control of the Rag2 promoter/enhancer (Rag2-GFP) [14,21]. Previous studies have shown that the expression of GFP from this transgene reflects endogenous Rag2 protein expression in lymphocytes, but with a longer half-life [21].

**Figure 6:**
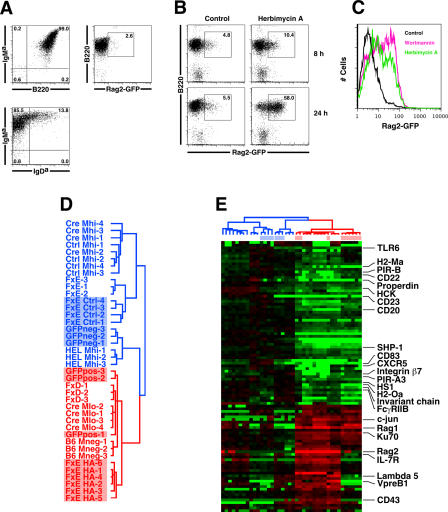
Immature B Cells Undergo a Reversal in Development after Blockade of Basal Ig Signaling with Herbimycin A or Wortmannin (A) Flow cytometric profile of HEL-Ig/Rag2-GFP BM at the end of a 5-d IL-7 culture. Cells are electronically gated by forward and side light scatter, and represent all lymphoid cells in the culture. The majority of cells were Rag-GFP^−^IgM^+^IgD^−^ immature B cells. (B) After a 5-d IL-7 culture, HEL-Ig/Rag2-GFP cells were washed and incubated with medium alone (containing DMSO carrier) or with 300 ng/ml herbimycin A for 8 or 24 h. Cells were then analyzed by flow cytometry, with cells gated based on size and annexin-V exclusion. Numbers represent percent of gated cells. (C) A representative experiment of Rag2-GFP expression levels in HEL-Ig/Rag2-GFP immature B cells incubated for 24 h with medium alone (black), herbimycin A (300 ng/ml) (green), or wortmannin (30 mM) (pink). (D and E) HEL-Ig/Rag2-GFP BM was cultured in IL-7 for 5 d, and cells were then incubated with herbimycin A (400 ng/ml) for 1 d. IgM^+^GFP^+^ [GFPpos (D)] cells were sorted from herbimycin A treated populations, and IgM^+^GFP^−^ [GFPneg (D)] cells were sorted from control cultures. In parallel, immature B cells were sorted from the BM of bcl-2 transgenic mice, and cultured with medium alone for 24 h (FxE Ctrl 1 and -2) or 48 h (FxE Ctrl 3 and 4), or with herbimycin A for 24 h (FxE HA-1, -2, -3, and -4) or 48 h (FxE HA-5 and -6). Total RNA was isolated from the cell populations and biotinylated cRNA probes were generated and hybridized to Affymetrix chips (E). All other cell populations were as described in Figure 2. The array data were clustered using the 101 transcripts from the Ctrl-M^hi^/Cre-M^lo^ gene list that were also differentially expressed between normal FxD (pre-B) and FxE (immature B) cells (using the following criteria: 2-fold or greater change in mean expression level, a greater than 200-unit difference in mean expression values, and Student's t-test *p* < 0.05). HC and LC transcripts were not used for this clustering analysis. The herbimycin A-treated immature B cells, both cultured and from bcl-2 Tg BM, cluster with more primitive cell populations. Data represent log_2_ fold-differences, with individual expression values for each gene divided by the mean expression value of the Ctrl-M^hi^, HEL-M^hi^, FxE, GFP^−^, and FxE^Ctrl^ populations. Representative genes are annotated.

Bone marrow from HEL-Ig/Rag2-GFP double transgenic (Tg) mice was cultured in IL-7 for 5 d, which generated a population of cells highly enriched for Rag2-GFP^−^, IgM^+^IgD^−^ immature B cells (Figure 6A). Cells were then washed and incubated with the tyrosine kinase inhibitor herbimycin A or medium alone for 8 or 24 h. Herbimycin A induced strong Rag2-GFP expression, with about half of the cells GFP^+^ at 24 h (Figure 6B). The observed GFP response was not due to nonspecific toxicity of herbimycin A treatment, since no GFP expression was observed in the dead or dying annexin-V^+^ or 7-AAD^+^ cells in the cultures (data not shown). Incubation of cells with other tyrosine kinase inhibitors (e.g., genistein and piceatannol) also resulted in significant induction of Rag2-GFP (L. E. Tze et al., unpublished data). BCR signaling and B cell development are highly dependent on phosphatidylinositol 3-kinase (PI3K) activity at the plasma membrane [22]. Incubation of HEL-Ig/Rag2-GFP immature B cells with the PI3K inhibitor wortmannin (30 μM) led to a strong induction of Rag expression at 24 h (Figure 6C). Thus, in a single cell assay for induction of Rag protein, interruption of proximal BCR signaling pathways with either herbimycin A or a PI3K inhibitor led to a derepression of Rag2 expression.

We then tested whether immature B cells treated with herbimycin A showed similar evidence for global back-differentiation as observed in Cre-M^lo^ cells. HEL-Ig/Rag2-GFP BM was grown in IL-7 for 5 d, and cells were washed and incubated either in medium alone or with herbimycin A for 24 h. Total RNA was extracted from sorted control Rag2-GFP^−^ (GFPneg in Figure 6D) and herbimycin-treated Rag2-GFP^+^ (GFPpos in Figure 6D) cells and examined by microarrays. As shown in Figure 6D and 6E, the gene expression patterns of Rag2-GFP^+^ cells coclustered with those of Cre-M^lo^, FxD, and B6-M^−^ cells, consistent with a reversal in development of immature B cells after blockade of signaling pathways with herbimycin A.

Next, we determined whether normal polyclonal immature B cells sorted from BM were capable of moving backward in development in response to loss of basal Ig signaling. This was important because it was possible that the ability of the cultured cells to back-differentiate might, in part, be a consequence of the accelerated development experienced by B cells carrying a pre-rearranged Tg Ig receptor [23]. Because of the increased sensitivity of immature B cells to apoptosis, we sorted immature B cells (B220^+^IgM^+^IgD^−^) to high purity (over 95%) from the BM of mice carrying a B cell-restricted *bcl-2* transgene [24]. These immature B cells were then cultured for 1 or 2 d either in medium alone (FxE Ctrl) or with herbimycin A (FxE HA), then total RNA was harvested for microarrays. Strikingly, polyclonal immature B cells treated with herbimycin A showed a reversal in development similar to that observed for the in vitro cultured Ig Tg B cells (Figure 6D and 6E), and clustered with cell populations that lacked an Ig receptor (B6-M^−^, FxD, Cre-M^lo^). The polyclonal immature B cells did not appear to back-differentiate to quite the same degree as the cells carrying a prerearranged BCR. For instance, *Rag* and *Ku70* genes were turned on in the polyclonal FxE cells in response to herbimycin A, similar to Cre-M^lo^ cells, while TdT, VpreB, and λ5 were not (Figure 6). We conclude that normal immature B cells undergo a reversal in development when treated with herbimycin A.

### New LC Rearrangements in Cells Undergoing Back-Differentiation

**Figure 7:**
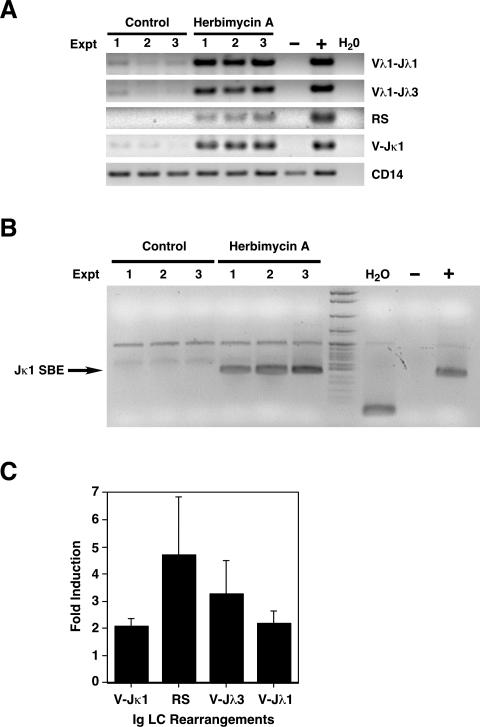
Immature B Cells That Lose Basal Signaling Show Induction of LC Rearrangements (A) PCR analysis of endogenous Ig light chain rearrangements (V-Jλ3, V-Jλ1, RS, and V-Jκ1) in genomic DNA of FACS-sorted HEL-Ig/Rag2-GFP BM cells incubated with medium alone or with 300 ng/ml herbimycin A for 24 h. IgM^a+^GFP^+^ cells were sorted from herbimycin A-treated cultures, and IgM^a+^GFP^−^ were sorted from control cultures. Data are from three independent experiments. CD14 is a loading control. −, negative control (C57Bl/6J tail DNA); +, positive control (C57Bl/6J spleen DNA). (B) Genomic DNA from the same cell populations described in (A) was subjected to ligation-mediated PCR to detect double-strand signal end DNA breaks at Jκ1. Controls in right three lanes of blot: H_2_O, control; −, negative control (S17 stroma); +, positive control (C57Bl/6 BM). (C) Genomic DNA was extracted from B1-8f/3-83κ and B1-8f/3-83κ/Mx-Cre immature B cells 3 d following incubation with IFNαβ. Quantitative PCR analysis was used to determine the fold-induction of LC rearrangements in B1-8f/3-83κ/Mx-Cre immature B cells treated with IFN compared to medium alone. Data represent the mean ± standard deviation of two (V-Jλ1), three (V-Jλ3), or four experiments (V-Jκ1, RS).

Finally, we wished to explore the possibility that cells losing basal Ig signaling and showing subsequent induction of Rag might also lose LC allelic exclusion. GFP^+^ cells were sorted from 24-h herbimycin A cultures of HEL-Ig/Rag2-GFP BM, and GFP^−^ cells were sorted from parallel control cultures. Genomic DNA was isolated and assayed for new endogenous Ig LC rearrangements using quantitative PCR. We observed strong induction of new DNA rearrangements at both κ and λ LC loci following incubation with herbimycin A (Figure 7A). In addition, double-stranded DNA signal end breaks, which are intermediates of active Rag-mediated recombination, were strongly induced in GFP^+^ herbimycin-treated cells (Figure 7B). Thus, the interruption of basal tyrosine kinase signaling pathways in immature B cells leads to the induction of the recombination machinery (e.g., Rag1 and Rag2) and new endogenous LC rearrangements.

Similar rearrangement assays were performed in cells undergoing Cre-mediated deletion of the B1-8f HC. We noted some “leakiness” of basal LC allelic exclusion in that culture system, with low levels of ongoing LC rearrangements even in the absence of deletion of the floxed Ig receptor (unpublished data). Nevertheless, cells losing BCR surface expression following Cre-mediated excision of the B1-8 HC showed a significant induction of LC rearrangements over background after 3 d of culture, ranging from 2- to 5-fold (Figure 7C). These data suggest that basal signaling through the Ig receptor in immature B cells is important for suppressing Rag gene expression and preventing further rearrangements at LC loci.

## Discussion

We report here the surprising result that inducible deletion of the BCR from immature B cells using Cre–lox-mediated excision leads to a global movement of cells to an earlier stage in B cell development. Similar findings were observed in immature B cells incubated with the tyrosine kinase inhibitor herbimycin A or the phosphatidylinositol 3-kinase inhibitor wortmannin. Our interpretation of these data is that immature B cells actively maintain their stage in development by constitutive basal Ig signaling through protein tyrosine kinases (PTKs).

Current models for signal transduction in lymphocytes envision a dynamic equilibrium between membrane-proximal protein tyrosine phosphatases (PTPs) and protein tyrosine kinases (PTKs) that serves to maintain cells in a resting state [25]. The BCR-associated molecules Igα and Igβ are phosphorylated on immunoreceptor tyrosine-based activation motifs (ITAMs) by the Src family PTK Lyn [26,27]. Phosphorylated ITAMs, in turn, lead to the recruitment and activation of the PTK Syk, which activates the Tec family PTK Btk. These kinases are counterbalanced by the activity of PTPs such as SHP-1 [28]. In the experiments presented here, incubation of immature B cells with the kinase inhibitors herbimycin A or wortmannin presumably shifts the equilibrium to one dominated by PTPs, resulting in an interruption of basal tyrosine kinase activity.

Loss of tonic BCR signaling in immature B cells, either through deletion of the BCR or incubation with kinase inhibitors, led to rapid changes in gene expression, with the overall picture suggesting a reversal in differentiation to an earlier stage in B cell development. It is intriguing that many transcription factors known to be important in controlling B cell development were modulated by the interruption of basal signaling. For example, the proto-oncogene myb, known to positively transactivate the Rag2 promoter [29], was up-regulated in Cre-M^lo^ cells, as was BLIMP, a transcriptional repressor that shuts off many B cell genes during terminal differentiation to plasma cells [30]. Oct-2, CIITA, and Egr-1, all known to transactivate B lineage genes, were strongly down-regulated in immature B cells following BCR deletion or treatment with herbimycin. Further dissection of these genetic pathways should provide new insights into the regulatory circuits that serve to actively maintain developmental stage in immature B cells.

A number of control experiments were performed to rule out the possibility that the back-differentiation observed could have been artifactual due either to a selective expansion of IgM^−^ cells present at the initiation of the cultures, or due to nonspecific effects of high-level Cre expression. First, in the Cre deletion experiments, cell counts, annexin V staining, and CFSE labeling demonstrated no evidence for a selective expansion or death of any subpopulation. Second, the Cre-M^hi^ population, in which approximately a third of the floxed alleles had been deleted, showed an intermediate gene expression phenotype between Ctrl-M^hi^ and Cre-M^lo^, indicating that the changes in gene expression were due to changes within the IgM^+^ population, since these cells were sorted based on being IgM^hi^. Third, flow cytometry showed that cell surface protein expression was similar across the entire population of cells for most of the markers, and no small subpopulations of cells were observed that could have contributed to the changes in gene expression profiles. Fourth, at the end of the some of the IL-7 cultures, B cells were isolated to high purity (more than 99% of cells B220^+^, IgM^interm/hi^) before treatment with IFN. After culture with IFN, these cells showed efficient BCR deletion and evidence for back-differentiation (see Figure S3). Finally, to rule out the possibility that nonspecific Cre-mediated DNA breaks at cryptic genomic loxP sites could be inducing DNA damage that was, in turn, responsible for the developmental changes observed, we showed that Cre recombinase had no effect on developmental status when expressed at high levels in control B cells lacking a floxed BCR (Figures S4 and S5). Taken together, these experiments point to a robust back-differentiation of immature B cells upon interruption of basal Ig signaling in this model system.

The Cre-M^hi^ cells still expressed surface IgM, yet showed early evidence for the back differentiation response (Table 1). One possibility to explain this finding is that Cre-M^hi^ cells have lowered levels of the BCR in intracellular compartments (e.g., endoplasmic reticulum), and that some portion of the basal signal derives from intracellular BCR complexes. Alternatively, and perhaps more likely, the basal level of signaling provided by the knock-in BCR in this model may be very close to the threshold required for suppressing Rag and the back-differentiation program, and flow cytometry is insufficiently sensitive to detect the small decreases in BCR surface expression levels that accompany the early loss of the floxed HC allele. In support of this, we note that Rag1 levels are nearly 10-fold higher in sorted Ctrl-M^hi^ cells (mean = 2,330 expression units) by microarray as compared to normal polyclonal immature B cells (FxE; mean = 243), and over 30-fold higher than conventional HEL-Ig Tg immature B cells (mean = 71). Similar differences were noted for Rag2. Suppression of endogenous LC rearrangements was also less efficient in the knock-in model as compared to the HEL system (Figure 7). Thus, these data are consistent with the notion that the B1-8f/3-83κ knock-in receptor provides a relatively weak basal signal, and that cells bearing these receptors are sensitive to very small changes in BCR density.

The idea that threshold signaling through the antigen receptor is required to suppress V(D)J recombination is supported by several observations in T and B cells. In cortical thymocytes, the interaction of the T cell receptor with MHC molecules down-regulates the expression of Rag1 and Rag2 and thereby shuts off further α chain rearrangements [31,32]. Interruption of this interaction with antibodies to the MHC resulted in up-regulation of *Rag* gene expression [33], indicating that the process is reversible in T cells. In an important series of experiments, Roose and colleagues [34] have recently shown that Jurkat T cells carrying mutations in proximal signaling molecules, including the adaptor molecules LAT and SLP76, have elevated Rag expression and altered gene expression programs. Using a panel of reconstituted Jurkat mutants and a variety of pharmacologic signaling inhibitors, the authors demonstrated that tonic signals through the ERK and Abl kinase pathways are important for suppressing Rag expression in Jurkat T cells and normal mouse thymocytes. B cells bearing a single copy of the anti-class I 3-83 HC and LC knock-in alleles showed poor LC allelic exclusion in mice lacking the cognate class I self-antigen (approximately 10% of B220^+^ cells idiotype-positive) [35,36]. Conversely, B cells from mice homozygous for both knock-in receptors (i.e., allelic inclusion) showed no evidence for additional LC rearrangements. One possibility is that the “dose” of receptor in mice carrying a single knock-in HC and LC allele did not provide sufficient basal signaling to suppress the recombination machinery, while two copies was sufficient. A similar Ag-receptor mediated suppression of Ig rearrangements was reported in mature IgD^+^ peripheral B cells treated with LPS and IL-4 [37]. These data support the hypothesis that developing B cells require threshold signals from Ig receptors expressed on the cell surface to block further Ig gene rearrangements.

The exquisite sensitivity of immature B cells to cell surface IgM basal signaling, as demonstrated by the early changes in gene expression in the Cre-M^hi^ population (Table 1), may be important for normal B cell development at several levels. First, this mechanism may serve as an important quality control mechanism. Cells that fail to rearrange a functional LC will continue to undergo rearrangements until an LC is produced that can be efficiently translated and stably expressed with HC at appropriate levels on the cell surface. This mechanism would also test the ability of HC and LC to pair efficiently and signal, ensuring that the B cell, once mature, can be activated in the periphery upon exposure to antigen. Second, this mechanism might be important for the selection of the naïve B cell V_H_/V_L_ repertoire [38]. V_L_ genes with strong promoters that drive high-level protein expression in immature B cells might be selectively recruited into the mature pool, while V_L_ genes driven by weaker promoters might fail to provide sufficient basal signals and would be replaced by secondary rearrangements. Similarly, particular individual V_H_ or V_L_ chains, or V_H_V_L_ pairs, with an enhanced ability to provide basal signaling might be relatively overrepresented in the repertoire.

Finally, we believe that these data have important implications for understanding the regulation of receptor editing in immature B cells. It has been suggested that self-Ag induced receptor editing is driven by the crosslinking of surface Ig on immature B cells, which activates Rag proteins and ultimately results in new LC rearrangements [5]. An alternative hypothesis is that cells triggered to undergo receptor editing may do so primarily in response to the down-regulation of Ig receptors following engagement of Ig with antigen. Our earlier data showing a reciprocal relationship between surface Ig density of immature B cells and receptor editing following Ig crosslinking by soluble antigen [15], and data demonstrating receptor internalization during receptor editing in immature thymocytes [39], are consistent with this idea.

We envision that some developing B cells at the immature stage will initially express high levels of the BCR, and only later during the immature stage encounter self-reactive antigens. These cells would receive a strong initial signal from self-antigen; however, the primary functional consequence of the self-antigen encounter would be the down-regulation of surface Ig via endocytosis. A sufficient loss of the basal signal would result in the re-initiation of Rag and induction of the back-differentiation program, which may be required for an efficient editing response. In support of this, we now have evidence that a back-differentiation program, similar to that observed in the models characterized by a loss of basal signaling, is initiated when immature B cells are incubated with specific antigen and undergo prolonged down-regulation of surface Ig (unpublished data). Together, these findings suggest that basal signals from the BCR control key developmental decisions at the pre-B to immature B cell transition.

## Materials and Methods

### 

#### Mice

Rag2-GFP transgenic mice [21] were kindly provided by Dr. M. Nussenzweig (Rockefeller University, New York, United States), and were bred with HEL-Ig transgenic mice [19] to generate double-Tg animals. The Mx-Cre transgenic [18], B1-8f [16] and 3-83κ [17] mice were intercrossed to generate B1-8f/3-83κ and B1-8f/3-83κ/Mx-Cre animals. Each mouse used in these experiments carried a single copy of the knock-in HC and LC Ig alleles. Additonal breedings produced HEL-Ig/Mx-Cre and B1-8f/3-83κ/Rag2-GFP animals. Bcl-2 mice [24] were obtained from Jackson Laboratories (Bar Harbor, Maine, United States). All mice were maintained in specific pathogen-free conditions, and were generally 4–8 wk of age at the time of the experiments.

#### IL-7 BM culture

Single-cell suspensions of BM cells from HEL-Ig/Rag2-GFP, B1-8f/3-83κ, and B1-8f/3-83κ/Mx-Cre mice were prepared as described [15], and placed into IL-7 BM culture [40,41,42]. Cells were cultured at a concentration of 1.5–2 × 10^6^ cells/ml in complete medium consisting of 1:1 RPMI 1640:EHAA (Mediatech, Washington, DC, United States, and Biofluids, Rockville, Maryland, United States, respectively), 10% heat-inactivated FBS (Life Technologies, Rockville, Maryland, United States), L-glutamine (BioWhittaker, Walkersville, Maryland, United States), penicillin and streptomycin (Mediatech), in the presence of 16 ng/ml recombinant murine IL-7 (R&D Systems, Minneapolis, Minnesota, United States) for 5–7 d. Cells were then washed and re-cultured in complete medium with either herbimycin A (Life Technologies) or wortmannin (Sigma, St. Louis, Missouri, United States) for 8 or 24 h, or with mouse fibroblast IFNαβ (Sigma) or recombinant IFNβ (Biosource, Camrillo, California, United States) for 1–4 d. For some experiments, IgM^hi^ cells were purified at the end of the IL-7 culture using the MACs bead system (Miltenyi Biotech, Bergisch Gladbach, Germany). Recombinant TAT-Cre was used as previously described [43]. For CFSE labeling, cells were incubated in 1× HBSS (BioWhittaker) and 3 μM CFSE (Molecular Probes, Eugene, Oregon, United States) for 5 min at 37 °C, washed, and then cultured in the presence or absence of IFNαβ for 1–4 d.

#### Flow cytometry and cell sorting

Cells harvested at the end of IL-7, herbimycin A, wortmannin, or IFNαβ cultures were stained in FACS buffer (PBS containing 2.5% FBS and 0.2% sodium azide) with FITC-, PE-, CYC-, APC-, or biotin-conjugated monoclonal antibodies to B220, IgM, IgM^a^, IgM^b^, IgD^a^, CD21/CD35, CD22, CD23, CD24, CD54, CD62 l, CD69, CD86, IL-7Rα, integrin β7, PirA/B, and IA/IE (BD Pharmingen, San Diego, California, United States) or 3-83κ (S27 mAb). Staining with biotinylated antibodies was revealed by SA-PE or SA-APC (BD Pharmingen). In some staining conditions, annexin-V-PE and/or 7-AAD (BD Pharmingen and Calbiochem, San Diego, California, United States) were used to exclude dead cells. For cell sorting, cells treated with IFNαβ or herbimycin A were stained with IgM^a^-PE and B220-CYC in staining buffer (PBS containing 10% FBS) and sorted by FACSVantage (Becton Dickinson, Mountain View, California, United States). Flow cytometry analyses were performed using CellQuest (Becton Dickinson) and Flowjo (Treestar, San Carlos, California, United States) software.

#### PCR analysis

Genomic DNA was extracted from unsorted BM cells treated with IFNαβ for 3 d as described [15,44], and from sorted BM cells treated with herbimycin A for 1 d or with IFNαβ for 2 d using TRIzol (Life Technologies). Genomic DNA from the equivalent of 40,000 cells, or serial dilutions thereof, was then subjected to quantitative PCR amplifications using primers specific for CD14, and degenerate Vκ primers and a primer downstream of Jκ1 as described [45,46], with slight modifications for the V-Jκ1 PCR conditions: 45 s at 94 °C, 1 min at 63 °C, and 1.5 min at 72 °C for 27 or 30 cycles [15]. RS recombinations were detected with the following primers: forward primers VDEG1, 5′- GCGAAGCTTCCCTGATCGCTTCACAGGCAGTGG-3′; VDEG2, 5′- GCGAAGCTTCCCW GCTCGCTTCAGTGGCAGTGG-3′; and VDEG3, 5′- GCGAAGCTTCCCAKM CAGGTTCAGTGGCAGTGG-3′; and reverse primerRS3′, 5′- CTCAAATCTGAGCTCAACTGC-3′. The following cycling conditions were used: 45 s at 94 °C, 1 min at 64 °C, and 1 min at 72 °C for 27 or 30 cycles [23]. V-Jλ rearrangements were detected with: forward primer, 5′- AGGCTGTTGTGACTCAGGAATCTGCA-3′, and reverse primer (JN) 5′- ACTTACCTAGGACAGTGA-3′ or (J16) 5′- ACTCACCTAGGACAGT-3′ using the following cycling conditions: 30 s at 94 °C, 1 min at 62 °C, and 1.5 min at 72 °C for 30, 33, or 36 cycles [47,48]. PCR products were resolved on 1% or 1.5% agarose gels and visualized with ethidium bromide staining. Specificity of bands was confirmed by Southern hybridization of nitrocellulose blots with radiolabeled internal oligonucleotides. Double-stranded signal end DNA breaks at Jκ1 were identified as described [49].

The extent of deletion of the B1-8f allele was quantified using a PCR-based assay to amplify across the 3′ loxP site. TRIzol-extracted genomic DNA (5 ng) from sorted cell populations was amplified with: forward primer, 5′- GAAAGTCCAGGCTGAGCAAAACACCAC-3′; and a reverse primer conjugated with 6-FAM (Integrated DNA Technologies, Coralville, Iowa, United States), 5′- GGAGACCAATAATCAGAGGGAAGAATAATA-3′. The cycling conditions were 15 cycles of 15 s at 95 °C, 30 s at 55 °C, and 1 min at 72 °C, followed by another 12 cycles of 15 s at 89 °C, 30 s at 55 °C, and 1 min at 72 °C. PCR product for the wild-type allele was 295 bp in length, while the product from the B1-8f allele was 378 bp. The extent of deletion was calculated by the loss of the B1-8f allele. These results were confirmed using the same reverse primer with a forward primer upstream of the 5′ loxP site. The assay was validated using Ctrl-M^hi^ cells (100% intact B1-8f allele) and Cre-M^lo^ cells (100% deleted B1-8f allele). PCR products were resolved by electrophoresis on a 3100 Genetic Analyzer with GeneScan-500 ROX size standards (Applied Biosystems, Foster City, California, United States). Quantitation of product intensities was performed using GeneScan Analysis 3.7 (Applied Biosystems).

#### Microarray gene chip analysis

Total RNA from sorted BM cells treated with IFNαβ or herbimycin A was extracted using TRIzol (Life Technologies) and further purified using an RNAeasy kit (Qiagen, Valencia, California, United States). Total RNA was then subjected to double-stranded cDNA synthesis following the standard Affymetrix protocol (Expression Analysis Technical Manual P/N 700218 rev. 2, Affymetrix, Santa Clara, California, United States). FxD and FxE samples were collected from independent sorts (each sample approximately 2 × 10^5^ cells) and total RNA extracted using TRIzol as described above. Total RNA was then subjected to two rounds of amplification to generate cRNA probes for hybridization, essentially as described [50]. Biotin-labeled cRNA probes (generated using High Yield RNA Transcript Labeling Kit, Enzo Diagnostics, Farmingdale, New York, United States) were chemically fragmented, hybridized to murine U74A and U74Av2 probe arrays (Affymetrix), and scanned at the University of Minnesota Biomedical Genomics Center facility. The U74Av2 probe arrays were subjected to U74A mask corrections, and all target hybridization intensities were scaled to 1,500 arbitrary units using Microarray Suite 5.0 (Affymetrix). Four independent sorts were performed for each of the following samples: Ctrl-M^hi^, Cre-M^hi^, and Cre-M^lo^; two or three sorts were performed for all other samples. Data with probe-set detection *p*-values less than 0.1 were included in the statistical analysis, and individual probe-set measurements with detection *p*-values greater than 0.1, and/or signal intensity values 20 or less, were set to a value of 20. We performed all our statistical analyses using MS Excel (Microsoft Office X). To identify genes that were differentially expressed between the Ctrl-M^hi^ and Cre-M^lo^ populations, we used the Student's t-test analysis with the assumptions that the sample groups followed a two-tailed distribution and had unequal variance. For visualization of the arrays, each expression value was divided by the mean of the expression values for the relevant IgM^+^ samples. These ratios were transformed into log_2_ space, and subjected to centered-average linkage clustering using CLUSTER and visualized by TREEVIEW software [51].

## Supporting Information

Figure S1 Cell Surface Profile of B1-8f/3-83κ and B1-8f/3-83κ/Mx-Cre B Cells B1-8f/3-83κ and B1-8f/3-83κ/Mx-Cre B cells were collected at the end of 5-d IL-7 cultures, stained with labeled mAbs, and analyzed by flow cytometry. Profiles are compared with wild-type B6 mature IgM^+^B220^hi^ splenic B cells, and the various compartments of B cells in BM. Second row, IgM^hi^B220^hi^ recirculating mature B cells; third row, IgM^+^B220^interm^ immature B cells; and fourth row, IgM^−^B220^lo^ pro- and pre-B cells).
(1.6 MB JPG).

Figure S2 Annexin V Staining of B1-8f/3-83κ and B1-8f/3-83κ/Mx-Cre B Cells After IFN Culture BM from control B1-8f/3-83κ and B1-8f/3-83κ/Mx-Cre mice was grown in IL-7 for 5 d. Cells were then washed, and treated with either medium alone or IFNαβ 1,000 units/ml for 1, 2, or 3 d. Cells were harvested and stained for B220, IgM^a^, and annexin V, and analyzed by flow cytometry. Shown are B220-gated cells. Similar results were obtained using 7-AAD in parallel to detect dead cells. Representative of five experiments.
(2 MB JPG).

Figure S3 Back-Differentiation of Highly Purified IgM^hi^ Cells To further address the issue of possible outgrowth or selective survival of IgM^lo^ cells present at the initiation of the IFN cultures, we isolated purified populations of IgM^hi^ (A) B1-8f/3-83κ and (B) B1-8f/3-83κ/Mx-Cre B cells at the end of the 5-d IL-7 cultures by first staining with predetermined optimal concentrations of biotinylated anti-IgM Abs, then isolating cells with streptavidin MACs beads. This reduced the IgM^lo^ population to approximately 0.6% of the B220^+^ cells prior to the secondary IFN cultures (compare postcolumn with precolumn flow profiles). Purified cell populations were incubated with either medium alone or 5,000 units of IFN for 2 d on irradiated S17 feeder cells, and then analyzed by flow cytometry. Numbers refer to percent of viable gated lymphocytes. IFN induced efficient Cre-mediated deletion of the BCR, and the IgM^lo^ cells in the cultures showed evidence for a reverse in differentiation as determined by elevated IL-7Rα expression and diminished CD22 expression levels. We noticed higher levels of deletion of the BCR in the medium-alone treated cells (in this experiment, 10.1%) compared with earlier experiments, likely due to crosslinking of IgM during the column purification step, and subsequent low level induction of Cre. There was approximately a 30% overall cell loss during the IFN culture and no cell proliferation (data not shown). We conclude that the IgM^lo^ cells present after the IFN culture began as immature IgM^hi^ cells. Representative of three experiments.
(369 KB JPG).

Figure S4 TAT-Cre Treated HEL-Ig B Cells Do Not Back-Differentiate To rule out the possibility that the back-differentiation observed was occurring in response to DNA damage due to high level Cre, we used a TAT-Cre fusion protein that was previously shown to induce Cre-mediated deletion of floxed alleles [43].
(A) In initial experiments, we performed titrations with the fusion protein and found dose-dependent deletion of the BCR at 24 h in B1-8f/3-83κ/Rag2-GFP immature B cells. In cells that lost the BCR, there was a significant induction of Rag2-GFP reporter activity, and up-regulation of IL-7Rα (data not shown). These data demonstrate the potency of the TAT-Cre preparation in this system and provide a single-cell analysis for the induction of Rag2-GFP following BCR deletion. Cell counts were similar in TAT-Cre and mock-treated cultures.
(B) The TAT-Cre fusion protein (200 μg/ml) was then introduced into HEL-Ig/Rag2-GFP immature B cells. Intracellular staining with anti-Cre antibodies in permeabilized cells confirmed that the TAT-Cre protein was introduced with good efficiency (note levels at both 2 and 24 h). B1-8f/3-83κ cells were tested in parallel for deletion of IgM (data not shown). Despite high intracellular levels of Cre, HEL-Ig immature B cells showed no evidence for back differentiation as determined by induction of Rag2-GFP, or up-regulation of IL-7R (data not shown). Representative of five independent experiments.
(437 KB JPG).

Figure S5 HEL-Ig/Mx-Cre Immature B Cells Fail to Back-Differentiate following IFN Treatment HEL-Ig mice were bred with Mx-Cre animals to generate HEL-Ig/Mx-Cre double transgenic mice. IL-7 BM cultures were established from two mice, and after 5 d IgM^+^ B cells were purified, washed, and then cultured for 2 d in 5,000 units/ml IFN. In parallel, a BM culture was established from a B1-8f/3-83κ/Mx-Cre mouse. After 2 d, cells were harvested, RNA was isolated, and cRNA probes were generated and hybridized to Affymetrix chips. Data analysis was performed identically to that described for Figure 2.
(A) CD22 levels were measured by flow cytometry for the various cell populations after IFN treatment.
(B) Shown are mean gene expression data for Ctrl-M^hi^ (*n* = 4) and Cre-M^lo^ (*n* = 4) samples for selected genes (see Figure 2 and Table 1). Fold differences were calculated as mean Cre-M^lo^/mean Ctrl-M^hi^. Also shown are the average raw data from the IFN-treated HEL-Ig/Mx-Cre samples (*n* = 2), and the parallel IFN-treated B1-8f/3-83κ/Mx-Cre sample. Fold differences were calculated as IFN-treated B1-8f/3-83κ/Mx-Cre/HEL-Ig/Mx-Cre. Overall, the gene expression patterns of the HEL-Ig/Mx-Cre samples closely mirrored the Ctrl-M^hi^ samples and showed no evidence for back-differentiation. In the same experiment, analysis of the cell surface profile and gene expression in HEL-Ig/Mx-Cre cells incubated with a PI3K inhibitor showed the expected back-differentiation response (data not shown), indicating that the HEL-Ig/Mx-Cre cells were capable of the response.
(C) Correlation coefficients were measured between the overall gene profiles of the two IFN-treated HEL-Ig/Mx-Cre samples compared with Ctrl-M^hi^ and Cre-M^lo^ mean values. Gene profiles of the IFN-treated HEL-Ig/Mx-Cre samples were significantly correlated with the Ctrl-M^hi^ samples (*r* = 0.655 and 0.637), and poorly correlated with the Cre-M^hi^ samples (*r* = 0.252 and 0.280), in the same range as Ctrl-M^hi^ vs. Cre-M^lo^ (*r* = 0.284).
(673 KB JPG).

### Accession Numbers

The Locuslink, or GeneID (www.ncbi.nlm.nih.gov/entrez/query.fcgi?db=gene, accession numbers of the genes and proteins discussed in this paper are λ5 (16136), Abelson murine leukemia (11350), B220 (19264), BLIMP (12142), Btk (12229), Burkitt lymphoma receptor 1/CXCR5 (12145), CD20 (12482), CD21/35 (12902), CD22 (12483), CD23 (14128), CD24 (12484), CD43 (20737), CD69 (12515) CD82 (12521), CD83 (12522), CD86 (12524), CD98 (20539), CIITA (12265), c-jun (16476), cyclin D2 (12444), cyclin-dependent kinase inhibitor 1a (12575), early growth response 1 (13653), elk4 (13714), Fcγ receptor IIB (14130), hemopoietic cell kinase (15162), ICAM-1/CD54 (15894), Igα (12518) and Igβ (15985), IGF2R (16004), IL-4 (16189), IL-7 (16196), IL-7R (16197), inositol 1,4,5-trisphosphate receptor (16438), integrin β7 (16421), Ku70 (14375), LAT (16797), LNK (16923), lymphoid enhancer factor 1 (16842), Lyn (17096), Mac-2 (16854), mitogen- and stress-activated protein kinase 2 (56613), mitogen-activated protein kinase (29857), MAP4K (26411), myb (17863), Notch1 (18128), Oct-2 (18987), paired-Ig-like receptor-A3 (18726), paired-Ig-like receptor-B (18733), phosphatidylinositol phosphate 5-kinase (18718), phosphodiesterase 7A (18583), PI3K (18708), protein kinase Cγ (18752), protein phosphatase 2Cβ (19043), *Rag1* (19373), *Rag2* (19374), serine-threonine kinase 3 (56274), sex determining region Y-box 4 (20677), SLP76 (3937), SH3-domain binding protein 2 (24055), SH3-domain binding protein 5 (24056), SHP-1 (15170), Son of Sevenless 2 (20663), Syk (20963), TdT (21673), thrombin receptor (14062), transforming growth factor β receptor (21812), Vav (22324), and VpreB (22362).

The microarray data have been deposited at GEO (http://www.ncbi.nlm.nih.gov/geo/; accession numbers are GSE2227.

## Abbreviations

7-AAD: 7-amino-actinomycin D
Ag: antigen
BCR: B cell receptor
BLIMP: B lymphocyte-induced maturation protein
BM: bone marrow
CFSE: 5(6)-carboxyfluorescein diacetate succinimidyl ester
CIITA: class II transactivator
Fx: fraction
GFP: green fluorescent protein
HC: heavy chain
HEL: hen egg lysozyme
IFN: interferon
Ig: immunoglobulin
IL-7: interleukin-7
IL-7R: IL-7 receptor
ITAM: immunoreceptor tyrosine-based activation motif
LC: light chain
MHC: major histocompatibility complex
PI3K: phosphatidylinositol 3-kinase
PTK: protein tyrosine kinase
PTP: protein tyrosine phosphatase
Rag: recombination activating gene
SHP-1: Src homology 2 phosphatase-1
TdT: terminal deoxynucleotidyl transferase
Tg: transgenic
